# Effect of comprehensive nursing intervention on the quality of life and clinical outcomes of patients with thyroid nodules treated by ultrasound-guided microwave ablation

**DOI:** 10.12669/pjms.40.4.7616

**Published:** 2024

**Authors:** Dan Chen, Fan Yang, Shao-ling Xing, Chun-zi Liu

**Affiliations:** 1 Dan Chen, Department of Oncology, Interventional Ultrasound, The Fifth Medical Center of Chinese PLA General Hospital, Beijing 100039, P. R. China; 2 Fan Yang, Department of Oncology, Seventh Ward, The Fifth Medical Center of Chinese PLA General Hospital, Beijing 100039, P. R. China; 3 Shao-ling Xing, Department of Anesthesiology, The Fifth Medical Center of Chinese PLA General Hospital, Beijing 100039, P. R. China; 4 Chun-zi Liu, Department of Oncology, The Fifth Medical Center of Chinese PLA General Hospital, Beijing 100039, P. R. China

**Keywords:** Comprehensive Nursing Intervention, Thyroid Nodule, Microwave Ablation, Quality of Life, Clinical Outcomes

## Abstract

**Objective:**

To evaluate the effects of comprehensive nursing intervention on the quality of life and clinical outcomes of patients with thyroid nodules treated by ultrasound-guided microwave ablation.

**Methods:**

This was a prospective study conducted from December 2020 to December 2022 at The Fifth Medical Center of Chinese PLA General Hospital. One hundred and twenty patients with benign thyroid nodules undergoing microwave ablation were included. Patients were randomly divided into control group and experimental group. Patients in the control group were given conventional intervention mode during the perioperative period, while those in the experimental group were given comprehensive nursing intervention mode on the basis of the control group. The differences in quality of life, cognitive level before and after intervention and satisfaction between the two groups were compared and analyzed.

**Results:**

The SF-36 scores in the experimental group were significantly higher than that in the control group after intervention. After the intervention, the SAS and SDS scores in the experimental group were significantly lower than those in the control group, with a statistically significant difference. The VAS scores in the experimental group were better than those in the control group at six, twelve and twenty four hour after operation, with statistically significant differences. After the intervention, the cognitive score of the experimental group was significantly higher than that of the control group.

**Conclusion:**

Comprehensive nursing intervention is worthy of clinical promotion in the treatment of patients with thyroid nodules treated by ultrasound-guided microwave ablation, leading to various benefits such as effectively improving patients’ quality of life and relieving pain.

## INTRODUCTION

Thyroid nodules, as a common disease in gland surgery, refer to scattered lesions caused by abnormal local growth of thyroid cells, with an increasing trend of incidence in recent years.^1^ Thyroid diseases are mainly goiter, hyperthyroidism, thyroiditis and thyroid tumor, and with the development of the course of the disease, thyroid function is abnormal, which will seriously affect the safety and quality of life of patients.^2^ The prevalence of thyroid nodules obtained by ultrasound examination is 20-70%, most of which are benign, while the prevalence of thyroid cancer is only 5-15%.^3^

Studies have shown that patients with thyroid nodules tend to be more anxious and depressed than healthy people^4^, and surgical treatment will aggravate negative emotions such as anxiety. Microwave ablation, as a new surgical treatment for thyroid diseases in recent years, is beneficial to the prognosis of patients with less postoperative trauma, but the trauma will still affect their mental health. Traditional perioperative care pays more attention to wound recovery, giving routine examination and service, but often lacks psychological care for patients. With the rapid development of nursing, the comprehensive nursing mode is also widely used in clinic. Comprehensive nursing mode is an intervention method based on continuous thinking, which has many advantages, such as personalization, integration and effectiveness.^5^

The purpose of this study was to analyze the influence of the comprehensive nursing intervention on the quality of life and therapeutic effect of patients with thyroid nodules treated by microwave ablation, so as to provide a reference for clinic.

## METHODS

This was a prospective study conducted at The Fifth Medical Center of Chinese PLA General Hospital from December 2020 to December 2022. One hundred and twenty patients with benign thyroid nodules undergoing microwave ablation were included and they were randomly divided into control group and experimental group, with 60 cases in each group. Patients in the experimental group were given comprehensive nursing intervention during the perioperative period, while those in the control group were given routine specialist nursing intervention. There was no significant difference in the general data between the two groups, which was comparable (Table-I).

**Table-I:** Comparative analysis of general data of the experimental group and the control group (*χ̅*±*S*) n=60.

Index	Experimental group	Control group	t/χ^2^	P
Age (years old)	68.73±8.82	69.25±8.47	0.33	0.74
Female (cases %)	39 (65%)	36 (60%)	0.32	0.57
Medical history (year)	2.74±0.58	2.68±0.42	0.65	0.52
BMI (kg/m^2^)	22.50±3.02	22.12±3.23	0.67	0.51
Nodule diameter (cm)	2.24±0.40	2.31±0.36	1.02	0.32
Nodule number			0.34	0.56
Single occurrence (cases %)	44 (73%)	47 (78%)	0.41	0.52
Multiple occurrences (cases %)	17 (27%)	13 (22%)		
Nodular nature				
Cystic (cases %)	21 (35%)	24 (40%)	0.32	0.57
Solid (cases %)	26 (43%)	20 (33%)	1.27	0.26
Cystic-solid (cases %)	13 (22%)	16 (27%)	0.41	0.52

p>0.05.

### Ethical Approval

It was approved by the Institutional Ethics Committee of The Fifth Medical Center of Chinese PLA General Hospital (No.: S2019-283-02; date: November 28, 2019), and written informed consent was obtained from all participants.

### Inclusion criteria:

- Patients diagnosed as benign thyroid nodules by puncture pathology.
- Patients with indications of microwave ablation.^6^
- Patients aged 50~75 years old.
- Patients who were conscious, had no mental disorder and could actively cooperate with the implementation of treatment and nursing plan.
- Patients with complete clinical data.
- Patients with informed consent and voluntary participation and have good treatment compliance.

### Exclusion criteria:

- Patients with serious diseases of other organs and systems at the same time
- Patients with a history of neck surgery.
- Patients with other important organs such as heart, liver and renal dysfunction.
- Patients with a severe mental disorder or cognitive dysfunction.
- Patients with poor treatment compliance and unable to cooperate with surgical treatment or nursing.

Patients in the control group were given conventional nursing mode after admission, i.e., conventional health education was given after admission to help patients get familiar with the hospital situation as soon as possible, take medicine according to the doctor’s advice and receive relevant treatment; Conventional preoperative education was given and complications were recorded in time to monitor patients’ vital signs, etc. Postoperative ice packs were applied intermittently to ameliorate swelling and pain in the neck tissue.

Patients in the experimental group were given a comprehensive nursing intervention mode after admission, including:

Pre-operatively, patients’ conditions were understood and personal files were established, including personal information such as age, educational background and past medical history. Nursing staff were required to pay attention to the cognitive reconstruction intervention of patients, introduce the pathogenesis, harmfulness and treatment methods of thyroid diseases to patients, explain in detail the treatment scheme and surgical prognosis of microwave ablation, correct patients’ wrong cognition of thyroid diseases and surgical treatment, strengthen patients’ confidence in the treatment effect; In addition, nursing staff were also required to explain the key points that patients need to cooperate in the treatment, such as how to communicate with the medical staff when swallowing and pain occurs. At the same time, nursing staff should create a good operating environment for patients.

Postoperatively, health education, including oral education, reading materials and video materials, were carried out in all directions to answer the questions of patients and their families, help patients find comfortable positions and guide them to exercise properly, to avoid the aggravation of pain caused by improper exercise, thus causing negative emotions; Nursing staff should pay close attention to the wound condition, change the dressing at the wound regularly to avoid incision infection, and focus on whether there are complications such as hoarseness, hematoma, neck urgency, etc.; When conditions permit, nursing staff should provide patients with a comfortable and quiet environment, divert patients’ attention by watching TV and other means according to patients’ condition and tolerance, and give analgesic drugs, analgesic pumps and other treatments when necessary to avoid negative emotions caused by pain; and help them successfully complete the transition from liquid food to soft food to a normal diet. If the upper laryngeal nerve is damaged, they should immediately fast and give energy supplements. Then, they should explain the mechanism of postoperative neck swelling to patients, and use the elastic bandage compression method and ice compress method to reduce neck swelling, observe closely when ice compress, and make regular reminders to prevent frostbite.

### Pre-discharge education

according to the treatment process of microwave ablation for benign thyroid nodules, the whole process of the segmented health education card set was carefully made, and a one-on-one oral presentation was made at the corresponding time link.

The nurse in charge and the patient exchanged WeChat before leaving the hospital, and after leaving the hospital, they took the initiative to provide continuous health guidance by phone + WeChat, so as to provide professional nursing support for rehabilitation outside the hospital. Mean follow up six months.

### Observation indexes:

### Comparative analysis of quality of life after intervention

The MOS Item Short from Health Survey (SF-36) were employed to evaluate and compare the quality of life of the two groups, including five dimensions: emotional function, role function, cognitive function, physical function and social function. The higher the score, the higher the quality of life.^7^

### Comparative analysis of anxiety and depression scores

Self-rating Anxiety Scale (SAS) and Self-rating Depression Scale (SDS)^8^ were used to evaluate the emotional changes of patients in the two groups before and after the intervention. The lower the score, the better the emotional state.

### Pain perception

Visual Analogue Scale (VAS)^9^ was used to evaluate the pain at 6 h, 12 h and 24 hours after the operation, with 10 being the perfect score, and the higher the score, the higher the pain level.

Comparative analysis of cognitive level before and after intervention: The cognitive level was evaluated with a self-made scale, including basic knowledge of diseases, matters needing attention after the operation, daily diet and matters needing attention during rehabilitation after discharge, with a total score of 100. The higher the score, the higher the cognitive level.

The Patient Satisfaction Questionnaire Short Form (PSQ-18)^10^ was used to compare and analyze the patients’ satisfaction before and after the intervention, including very satisfied, relatively satisfied, satisfied, uncertain and dissatisfied.

Total satisfaction= (very satisfied+relatively satisfied+satisfied)/total number of cases x100%.

In this study, all the questionnaire were given to the participants for investigation, and then collected in about five minutes.

### Statistical analysis

All the data in this study were statistically analyzed by SPSS 20.0 software, and the measurement data were expressed in (*χ̅*±*S*). A T-test of two independent samples was used for data analysis between groups, paired t-test or variance analysis was used for data analysis within groups, and c^2^ was used for rate comparison. The confidence interval is 95%. p<0.05 indicates a statistically significant difference.

## RESULTS

After the intervention, the scores of emotional functions, role function, cognitive function, physical function and social function in the experimental group were significantly higher than those in the control group, with statistically significant differences (*p=*0.00) (Table-II).

**Table-II:** Comparative analysis of quality of life scores between the two groups before and after intervention (*χ̅* ±*S*) n=60.

Group	Emotional function	Cognitive function	Somatic function	Role function	Social function
Experimental group	7.18±1.32	5.06±0.78	8.40±1.67	5.82±0.73	4.06±1.21
Control group	6.23±1.46	3.65±0.70	6.06±1.35	4.31±0.34	2.38±0.86
*t*	3.74	10.42	8.44	14.52	8.73
*p*	0.00	0.00	0.00	0.00	0.00

*p<0.05.

No statistically significant difference was observed in SAS and SDS levels between the two groups before the intervention (p>0.05). After the intervention, the SAS and SDS scores in the experimental group were significantly lower than those in the control group, with a statistically significant difference (p=0.00) (Table-III).

**Table-III:** Comparative analysis of the emotional status of patients in the two groups before and after intervention (*χ̅* ±*S*) n=60.

Index		Experimental group*	Control group	t	p
SAS	Before intervention	63.21±6.37	62.82±6.15	0.34	0.73
	After intervention*	45.25±6.81	51.53±6.71	5.10	0.00
SDS	Before intervention	54.58±5.70	54.93±5.26	0.35	0.73
	After intervention*	41.39±5.01	46.82±5.40	5.71	0.00

* p<0.05.

The comparison of pain between the two groups at six hours, 12 hours and 24 hours after the operation (Table-IV) indicated that after the intervention, the VAS scores in the two groups gradually decreased with time, with statistically significant differences (p=0.00); The VAS scores in the experimental group were better than those in the control group at six hours, 12 hours and 24 hours after operation, with statistically significant differences (p=0.00).

**Table-IV:** Comparative analysis of VAS scores between the two groups (*χ̅* ±*S*) n=60.

Group	6h after operation	12h after operation	24h after operation	F	p
Experimental group	3.47±0.32	2.03±0.13	0.78±0.02	18.65	0.00
Control group	4.62±0.15	3.12±0.17	1.10±0.07	17.46	0.00
*t*	25.21	42.53	34.05		
*p*	0.00	0.00	0.00		

p<0.05.

No statistically significant difference was observed in cognitive scores between the two groups before the intervention (p=0.66). After the intervention, the cognitive score of the experimental group was significantly higher than that of the control group, with a statistically significant difference (p=0.00) (Table-V). The satisfaction level in the experimental group was 100%, which was significantly higher than 87% in the control group, with a statistically significant difference (p=0.00), (Table-VI).

**Table-V:** Comparative analysis of cognitive level between the two groups before and after intervention (*χ̅* ±*S*) n=40.

Group	Before intervention	After intervention*
Experimental group	46.23±12.42	79.51±12.40
Control group	45.79±13.16	72.44±11.37
*t*	0.47	3.43
*P*	0.66	0.00

* p<0.05.

**Table-VI:** Comparative analysis of satisfaction rate between the two groups (*χ̅* ±*S*) n=60.

Group	Very satisfied	Relatively satisfied	Satisfied	Uncertain	Dissatisfied	Total satisfaction rate*
Experimental group	34	11	15	0	0	60 (100%)
Control group	33	13	6	5	3	52 (87%)
c^2^						8.57
P						0.00

* p<0.05.

## DISCUSSION

This study showed that the SAS score for anxiety and SDS score for depression, the VAS score for postoperative pain and SF-36 in the experimental group were significantly better than those in the control group (p<0.05), suggesting that comprehensive nursing intervention in patients with thyroid nodules is beneficial to reduce pain perception, reduce negative emotions and improve quality of life. This may be attributed to the fact that the intervention method added targeted detail nursing to the traditional nursing scheme, and carried out psychological nursing based on patients’ mental health, so as to achieve all-round intervention in time, form and content.^11^

A thyroid nodule is a common head and neck surgical disease with the development of the disease course, it may affect patients’ respiratory function and swallowing, and may lead to thyroid dysfunction and canceration^12^ with the development of minimally invasive and imaging technology in recent years, microwave ablation, as a tumor treatment ablation technology, has emerged, and it has been widely used in benign and malignant thyroid diseases.^13^ It has been clinically proved that its curative effect is ideal and it is safe at the same time. Internationally, microwave ablation (MWA) is one of the most widely used thermal ablation techniques^14^, resulting in various advantages such as small wounds, being beneficial to the prognosis of patients, leaving almost no scar, and the psychological damage to patients is far lower than that of traditional thyroidectomy. It is urgent to explore scientific and effective intervention methods to ameliorate the current situation in clinic. Reasonable intervention methods can not only improve patients’ negative emotions but also improve their compliance, which is conducive to improving the treatment effect.

Comprehensive nursing intervention is a new type of nursing method, which aims at a certain disease combined with the specific situation of patients and carries out nursing according to a predetermined method.^15^ Compared with traditional nursing, it is more operational and easy for patients to accept, and it is a more practical and perfect nursing model^16^ that pays more attention to the psychological care and comfort of patients.^17^Comprehensive nursing intervention emphasizes providing patients with all-round, detailed and high-quality services before and after the operation, improving patients’ bad emotional state through psychological counseling before the operation, strengthening preventive nursing of complications after the operation, closely monitoring the changes of signs, understanding the changes of illness, providing dietary guidance and health education, and improving nursing effect.

Detailed comprehensive nursing is very beneficial. It can not only reduce the degree of pain caused by diseases and operations, relieve patients’ nervousness and anxiety, and improve their quality of life, but also significantly improve patients’ experience of seeing a doctor, and increase their satisfaction and their awareness of diseases. The comprehensive nursing mode provides all-round guidance according to patients’ conditions and postoperative situation, formulates a rehabilitation plan in line with patients’ conditions and a follow-up treatment plan after discharge, corrects their past wrong habits and concepts, thus reducing related adverse reactions.^18^ After the intervention, the patients had less postoperative pain, good wound condition and low discomfort, which further reduced the generation of negative emotions, and had greater trust in the treatment plan and medical staff and social satisfaction.

Chandratre et al^19^ considered that a comprehensive nursing intervention mode could increase the relationship between nurses and patients, improve patients’ psychological satisfaction and sense of security, and enhance their confidence in overcoming diseases so that patients can actively cooperate with medical staff to receive treatment, which is conducive to the recovery of their illness. Evidence shows that^20^ comprehensive nursing has certain advantages in improving patients’ nursing experience and treatment compliance, and adopting a warm and cordial attitude to contact with patients can improve patients’ compliance. It was also confirmed in our study that the cognitive score of patients after the comprehensive nursing intervention was significantly higher than that of patients receiving conventional nursing mode (p=0.00). And patient satisfaction was as high as 100%, which was significantly higher than that of the control group (p=0.00).

### Limitations

It includes small sample and the follow-up time was short. In order to cope with this, the sample size will be further expanded and the follow-up time will be extended in the future clinical work, with a view to evaluating the advantages and disadvantages of the intervention scheme more objectively and benefit more patients.

## CONCLUSIONS

The comprehensive nursing intervention has a very positive effect on the psychological status of patients with thyroid nodules after microwave ablation, which is conducive to reducing pain perception, improving anxiety and depression, enhancing their compliance and satisfaction, and improving the treatment effect and prognosis.

### Authors’ Contributions:

**DC** and **FY:** Designed this study, prepared this manuscript, are responsible, accountable for the accuracy and integrity of the work.

**SX:** Collected and analyzed clinical data.

**CL:** Participated in acquisition, interpretation of data and draft the manuscript.

All authors read and approved the final manuscript.

## References

[1] Wong R, Farrell SG, Grossmann M (2018). Thyroid nodules:Diagnosis and management. Med J Aust.

[2] Durante C, Grani G, Lamartina L, Filetti S, Mandel SJ, Cooper DS (2018). The Diagnosis and Management of Thyroid Nodules: A Review published correction appears in JAMA. 2018;319(15):1622. JAMA.

[3] Xue SW, Luo YK, Jiao ZY, Xu L (2021). Clinical value of SMI Combined with Low-Dose CT Scanning in differential diagnosis of Thyroid Lesions and Tumor Staging. Pak J Med Sci.

[4] Dreger S, Pfinder M, Christianson L, Lhachimi SK, Zeeb H (2015). The effects of iodine blocking following nuclear accidents on thyroid cancer, hypothyroidism, and benign thyroid nodules:design of a systematic review. Syst Rev.

[5] Coppell KJ, Abel SL, Freer T, Gray A, Sharp K, Norton JK (2017). The effectiveness of a primary care nursing-led dietary intervention for prediabetes:a mixed methods pilot study. BMC Fam Pract.

[6] Papini E, Monpeyssen H, Frasoldati A, Hegedüs L (2020). 2020 European Thyroid Association Clinical Practice Guideline for the Use of Image-Guided Ablation in Benign Thyroid Nodules. Eur Thyroid J.

[7] Lončar I, Van Dijk SPJ, Van Velsen EFS, Buijk SE, Niemeijer ND, Veeken CJ (2022). Radiofrequency Ablation for Benign Symptomatic Thyroid Nodules in the Netherlands:Successful Introduction of a Minimally Invasive Treatment Option Improving Quality of Life. J Vasc Interv Radiol.

[8] Yue T, Li Q, Wang R, Liu Z, Guo M, Bai F (2020). Comparison of Hospital Anxiety and Depression Scale (HADS) and Zung Self-Rating Anxiety/Depression Scale (SAS/SDS) in Evaluating Anxiety and Depression in Patients with Psoriatic Arthritis. Dermatology.

[9] Sung YT, Wu JS (2018). The Visual Analogue Scale for Rating, Ranking and Paired-Comparison (VAS-RRP):A new technique for psychological measurement. Behav Res Methods.

[10] Thayaparan AJ, Mahdi E (2013). The Patient Satisfaction Questionnaire Short Form (PSQ-18) as an adaptable, reliable, and validated tool for use in various settings. Med Educ Online.

[11] Satherley RM, Lingam R, Green J, Wolfe I (2021). Integrated health Services for Children:a qualitative study of family perspectives. BMC Health Serv Res.

[12] Cheng Z, Liang P (2020). Advances in ultrasound-guided thermal ablation for symptomatic benign thyroid nodules. Adv Clin Exp Med.

[13] Orloff LA, Noel JE, Stack BC, Russell MD, Angelos P, Baek JH (2022). Radiofrequency ablation and related ultrasound-guided ablation technologies for treatment of benign and malignant thyroid disease:An international multidisciplinary consensus statement of the American Head and Neck Society Endocrine Surgery Section with the Asia Pacific Society of Thyroid Surgery, Associazione Medici Endocrinologi, British Association of Endocrine and Thyroid Surgeons, European Thyroid Association, Italian Society of Endocrine Surgery Units, Korean Society of Thyroid Radiology, Latin American Thyroid Society, and Thyroid Nodules Therapies Association. Head Neck.

[14] Tufano RP, Pace-Asciak P, Russell JO, Suárez C, Randolph GW, López F (2021). Update of Radiofrequency Ablation for Treating Benign and Malignant Thyroid Nodules. The Future Is Now. Front Endocrinol (Lausanne).

[15] Eastwood J, Maitland-Scott I (2020). Patient Privacy and Integrated Care:The Multidisciplinary Health Care Team. Int J Integr Care.

[16] Bardin T, Chalès G, Pascart T, Flipo RM, Korng Ea H, Roujeau JC (2016). Risk of cutaneous adverse events with febuxostat treatment in patients with skin reaction to allopurinol. A retrospective, hospital-based study of 101 patients with consecutive allopurinol and febuxostat treatment. Joint Bone Spine.

[17] Kaul S, Gupta M, Bandyopadhyay D, Hajra A, Deedwania P, Roddy E (2021). Gout Pharmacotherapy in Cardiovascular Diseases:A Review of Utility and Outcomes. Am J Cardiovasc Drugs.

[18] Coppell KJ, Abel SL, Freer T, Gray A, Sharp K, Norton JK (2017). The effectiveness of a primary care nursing-led dietary intervention for prediabetes:a mixed methods pilot study. BMC Fam Pract.

[19] Chandratre P, Mallen C, Richardson J, Muller S, Hider S, Rome K (2018). Health-related quality of life in gout in primary care: Baseline findings from a cohort study published correction appears in Semin Arthritis Rheum. 2022;52:151800. Semin Arthritis Rheum.

[20] Chen MQ, Wang HY, Shi WR, Sun YX (2021). Estimate of prevalent hyperuricemia by systemic inflammation response index:results from a rural Chinese population. Postgrad Med.

